# Medical Items and News of Interest to the Physician

**Published:** 1879-04

**Authors:** 


### For the Use of Physicians.

CULLED FROM THE STANDARD MEDICAL JOURNALS OF THE WORLD.

NOTE.—These formulæ are intended only for the regular practitioner of medicine, and should be employed only by him, or under his immediate supervision.

**Ether Jelly.**

Mix the white of one egg with one troy ounce and a half of sulphuric ether, and shake briskly.

**Sulphurous Acid as a Remedy for Puritus.**

The editor of the *Obstetric Gazette* reports cases of puritus of the vulva and accompanying eczema which have been at once relieved by using as a lotion sulphurious acid in full strength.

**Ammonio-Sulphate of Copper in Epileptiform Facial Neuralgia.**

Frere, of the Lariboisiere, has found this, in doses of 0.07 twice a day, succeed well when other means had failed. In one case 0.10 was given at one dose.—*Richmond and Louisville Medical Journal.*

**Curare for Epilepsy.**

Kunze has lately recommended curare to be employed in the form of a subcutaneous injection at intervals of about a week. The formula he adopts is seven grains of curare, dissolved in seventy-five minims of water mixed with two drops of muriatic acid, eight drops of this being a dose.

**Compound Bismuth Powder.**

Subnitrate of bismuth, 12 grains.
Powdered ipecac, 2 grains.
Carbonate of magnesia, 2 drachms.
Oil of anise, 1 drop.

Mix, and divide into twelve powders. Some omit the essential oil. The dose is one powder, two or three times a day, in gastrodynia.

**Morphia Hypodermically in Dysentery.**

Dr. J. E. Washington reports that he has found hypodermic injections of about $\frac{1}{8}$ grain of morphia to be a most certain remedy for dysentery accompanied with tenesmus. It appears to act like magic. Its rapid effects cannot be produced by opium.—*Nash. Journ. of Med.*

**Treatment of Cancer by Preparations of Guaco.**

(G. v. Schmidt: *Chi. f. Chirurgie* 1878, No. 16; from *Wein. Med. Wochenschrift.*) There seems no reason *a priori* why some internal remedy may not be discovered which shall do for epithelial and other new growths what iodine does for syphilitic new formations. Dr. Schmidt suggests guaco, which he has used in the form of tincture and plaster.

**Sarracenia Purpurea in Gout.**

According to Foucaut the stem and root of Sarracenia purpurea is an effective remedy in chronic form of gout. It is employed in form of an infusion, made from 1 to 2 spoonfuls of the powder at a time, mornings and evenings, and once during the day 1 spoonful made into an infusion as a prophylactic. The violence of the attacks becomes greatly diminished, and the alveolar movements more regular.—*Arch. der Med. in Ph. Zeit. f. Russel.*

**Apomorphia as an Expectorant.**

Drawing a conclusion from the fact that ordinary emetics, when administered in small doses, generally act as expectorants, the director of the Heidelberg Polyclinic made experiments with small doses of apomorphia. It was found to greatly facilitate expectoration, and to quickly remove dry and sibilant rales. It was given in doses of $\frac{1}{64}$ to $\frac{1}{30}$ grain in solution, the hydrochlorate being preferred.—*Rev. Therap. Med.-Chir.*

**Inhalation of Carbolic Acid Spray in Phthisis.**

At the Mt. Sinai Hospital, New York City, the inhalation of carbolic acid spray in phthisis has been introduced, in order to test its efficacy. The spay was obtained from a solution holding two per cent. of the acid. The first case had fetid expectoration, with an average temperature of $102\frac{1}{2}°$. The first effect on the inhalation was to increase the sputa to a marked extent, but at the same time to check the fetor. The most important effect of the inhalation was to decrease the temperature from $102\frac{1}{2}°$ to $101°$, $100\frac{1}{2}°$ and $99°$. In some of the cases, carbolic acid acted as an irritant, giving rise to considerable spasmodic effects, and in these cases salicylic acid was substituted. The latter agent did not produce such a decided effect on the temperature, but its action on the fetor was equally marked.—*Phil. Med. and Surg. Rep.*

**Iodoform.**

Finely powdered iodoform, or mixed one part to three with unguentum petrolei, makes an admirable application to the most sensitive surface, such as irritable ulcers, etc. It is a good rule never to apply soap to such surfaces; even the best obtainable is often irritating; and water should be thoroughly boiled and used when cooling. To do away the odor of the iodoform, so nauseous to many, it may be mixed with equal parts of tanin, or employed in ethereal solution.—*Med. and Surg. Rep.*

---

**Syrup of Croton Chloral Hydrate,**

Containing 2 grs. in each fluid drachm, may be made by dissolving 16 grs. of croton chloral hydrate in 30 minims of absolute alcohol, or a drachm of rectified spirit, and adding syrup of any desired flavor, sufficient to make an ounce.

The following yields a nicely flavored syrup:

℞

Crot. chlor. hyd., gr. xvj.

Tinct. aurant. recent, ʒj.

Solve et adde

Syrupi, ʒvij.

---

**Senecio Arvensis as a Febrifuge.**

This plant, known by the common name of groundwort, is said by a Flemish physician to be superior to cinchona as a febrifuge. Eight hundred grains of the fresh herb, excluding the root, are to be boiled for ten minutes in a pint of water, and the decoction then strained. This is to be taken in three doses at intervals of two hours after the attack. In nearly every case relief or cure is the result.

The senecio arvensis is perhaps not a native of the United States, but it is likely that S. aureus, or squaw weed, or common groundsel, might be its equivalent.

---

**Destruction of Oxyures.**

Mr. Laboulbene recommends a new method for destroying the oxyures or small thread-like worms infesting the anal folds, causing such insupportable itching, and which are so difficult to get rid of. This means consists in adjections of syrup (or molasses) or glycerin. Every oxyures touched by these liquids soon disappears, and as there is nothing irritant about them, they can be repeated often enough to exterminate every adult, and consequently destroy the race as soon as the eggs previously deposited shall have been destroyed.—*Canad. Jour. of Med. S.*

**Elastic Crayons of Nitrate of Silver.**

Dr. Pajot takes a laminaria-tent, two millimeters (about $\frac{1}{10}$ inch) in thickness, dips it into thick mucilage, and rolls it into finely powdered lunar caustic. When it dries, it forms a crayon, of the usual thickness of a stick of nitrate of silver, which can be introduced into the cavity of the uterus without fear of breakage. In the same manner, applications can be made to other cavities, and, if necessary, without stronger remedies.—*All. Med. Zeit.*

---

**Strichnia as an Anti-Emetic.**

M. Empis (of LaCharite, Paris), uses a very dilute alcoholic solution of strychnia for allaying that kind of vomiting which is called "nervous," because it does not correspond to any organic lesion. Although there are quite a number of such emetics, still the strychnia solution is highly praised by Mr. Empis. He directs the following mixture to be boiled for some minutes: strychnie, 1 centigram ($\frac{3}{20}$ths of a grain); alcohol, 1 gram; water, 99 grams; then to throw the whole on a filter of paper, which retains the undissolved strychnia. The liquid which passes through possesses a very great bitterness. M. Empis usually gives this liquid in the dose of three tablespoonfuls in the day. It is not unusual to see the vomiting stop after the first.—*Canad. Jour. of Med. S.*

---

**Carbazotate of Ammonium in Treatment of Malerial Fever.**

J. J. Schefferstein, of Olney., Ill, failed to obtain beneficial results in the use of carbazotate of ammonium in malarial fevers, when he employed in cases that had been suffering for some months or years from malarial poisoning. When, however, he preceded its use by the exhibition of saline purgatives, diaphoretics, and chalybeate tonics for several (twelve to fourteen) days, it seldom failed. He advocates the use of the remedy in small doses, and in combination with other remedies, such as arsenic and iron. One-fourth to one grain may be used for a dose, and may best be given in pill form repeated thrice daily for three to six days. In doses of $\frac{1}{16}$ gr., it may be continued two to three weeks. The experience of this writer in seven hundred cases shows that, while the carbazotate of ammonium is not so useful in breaking up an attack of malarial fever as quinia, it protects the system longer from a recurrence, and is, he believes, the *sine qua non* of all remedies for "chronic chills."—*Illinois Medical Recorder.*

**Thymol and its Uses.**

Dr. B. Kussner, of Halle, has tried thymol internally. In doses of three to five drops of a one-per-cent solution he found it useful in the diarrhœa of children. An inhalation of one part to one thousand of water reduced the fever and expectoration in a phthisical case.

Animals poisoned with thymol sink into a profound coma. After death their blood is dark and fluid. Fatty degenerations of internal organs is, however, not found. Injected into the veins, it lowers the temperature and induces stupor.—*Med. Press and Circ.*

**A Nervine and Antispasmodic.**

A favorite prescription in the Hospital of Chest Diseases, London, is the following, useful in epilepsy, chorea, dysmenorrhœa, hysteria epileptica, and like nervous conditions :

℞

Potassii bromidi gr. x.

Tinct. conni ♏ xxx.

Tinct. valerianæ ammon ♏ xx.

Aquæ camphoræ fl ℥ i.

For one dose, thrice daily.

**Villates Mixture in the Treatment of Sinuses.**

In several deep sinuses under treatment in Charity Hospital, N. Y., in which no necrosed bone could be found, but which proved intractable to heal, Villates mixture was injected, first of half strength and then full strength, once a day. The following is the composition of the mixture :

℞

Liq. plumbi subacet ʒ i.

Zinci sulph. cryst.,

Cupri sulph. cryst āā ʒ ss.

Aceti vini albi ℥ viss.

Mix.

**Treatment of Diphtheria.**

M. Kien, in the late epidemic of diptheria at Strasburg, has found that Schaller's method of treating diphtheria with perchloride of iron—twenty drops in twenty drops of water, in a teaspoonful or two of coffee every two hours, was exceedingly effective. In some cases, in which the medicine did not act sufficiently rapidly, M. Kien has given in addition Syrup of Eucalyptus, according to the plan of M. Goldschmidt. If the patients refused to take the perchloride of iron, a lotion was employed, such as was proposed by M. Mandl, of Paris, for application in chronic granular pharyngitis. The lotion was applied by means of a brush, as a wash for the sore places, two or three times a day. It was composed of carbolic acid 0.10, pure iodine, potassium, iodide, āā. 0.20; glycerin, 10.00 grams. Independently of this, he gives salicylate of sodium, if symptoms of fever present themselves; the drug acts in the same way as sulphate of quina, while it is more easy to administer in a liquid form.—*Gazette Medicale de Strasburg*, in *The Practitioner.*

**Nitrite of Amyl and Chloroform.**

Prof. L. B. Ballic (*in Med. and Surg. Rep.*) claims great immunity from the dangers of asphyxia in chloroformic anæsthesia, if the anæsthetic is combined and administered with nitrite of amyl, in proportion of 15 drops to the ounce. His large experience, as well as theory, bears him out in this important discovery.—*Rich. and Louisv. Med. Journal.*

The *Deutsche Medic. Wochenschrift*, 1878, No. 45, contains the full account of another case, in which the inhalation of a few drops of nitrite of amyl apparently restored the patient from threatening asphyxia by chloroform narcosis, after artificial respiration had proved ineffectual.—*Lancet and Clinic.*

**The Oil of Amber in Anginose Affections,**

Dr. A. R. Fink, of Philadelphia, recommends very highly the oil of amber for the relief of the neuralgic element of anginose affections. In support of this he cites several cases treated by this drug with marked success. It was only used in conjunction with other treatment, but the pain ceased so invariably on the use of the amber, and in several cases, reappeared so quickly on its disuse, that, in Dr. Fink's opinion, there could be no question as to the casual relation of the administration of the amber to the relief of the pain. It is the presence of neuralgic pains, and not the organic or functual character of the heart affection, that indicates the use of this agent. Hysterical angina frequently receives the happiest relief from the addition of from eight to ten drops of the oil as an antihysterical dose, repeated every twenty minutes. *Medical Times.*

**Condurango Heard From Again.**

In the *Deutsche Med. Worch.* Dr. Burkmann relates two cases of cancer of the stomach, so diagnosed by several skillful physicians, both very much relieved, and one quite cured, by the use of condurango bark, according to the form and dose recommended by Prof. Friedreich, (described in Naphey's *Surgical Therapeutics*, p. 464). Dr. Burkmann earnestly recommends this plan to his colleagues. He is careful, during the treatment, to forbid the use of opium or morphia. His words certainly deserve attention, for we are powerless before this terrible disease.—*Med. and Surg. Rep.*

---

**Arenaria Ruba in Gravel and Catarrh of the Bladder.**

(E. L. Bertherand.) *Arenaria ruba* is a very common plant in the environs of Algiers and in the neighboring districts. As it prefers a sandy soil, it is vulgarly called *sabline*. This plant is much used in Malta and Sicily by physicians in the treatment of vesical catarrh, and even gravel. The author found, on trial, that it is a most effective remedy in these complaints; he also noticed the remarkable fact that, after its use, the peculiar putrid ammonical, and often intolerable odor of urine of such patients is entirely removed. The best method of administration is a decoction of the dried plant.—*Jour. de Ph. et Ch.*

---

**Slippery-Elm Bark for Tape-Worm.**

Dr. C. Hixon, in the *Ohio Medical Recorder*, says: "I was consulted by Mr. J., who at various times had passed large quantities of tape-worm. I prescribed for him the ethereal oil of male fern, also kousso, kamala, and pumpkin seed without any result. I then prescribed fresh elm bark *ad libitum*, of which for several days he consumed large quantities. I then gave him castor oil and turpentine emulsion; when fifty-three feet of tape-worm were expelled, including the head. It was enveloped in the great mass of apparently undigested bark, which the cathartic brought away. Becoming entangled in it the worm seems to have lost its grip and never again regained it, and was hurried along by the increased peristaltic action."—*Louisville Medical News.*

---

**Hydrobromate of Quinia as an Antipyretic.**

Dr. Esquerdo administrated this salt to a typhoid patient, whose temperature had been 40° C. for several days. Two days afterwards the temperature was reduced to 36° C. At this point, as the author feared collapse, since the reduction had been so considerable, the hydrobromate of quinia was no longer given; the temperature became higher. In two cases of phthisis the hydrobromate caused a disappearance of the feverish spmptoms and swellings.—The dominant action of hydrobromate of quinia consists in its power of causing a lowering temperature and of diminishing the frequency of the pulse while it increases its amplitude. The observations of Dr. Esquerdo are corroborated by Dr. Cahis, who has observed in a typhoid patient a lowering of temperature from 41° to 39° or even to 38° C., brought on by the administration of fifty to seventy centigrams (= 8 to 11 grains) of the hydrobromic salt.—*Independenica Medica* in *The Practicioner.*

---

**Expulsion of Tape-Worm.**

Although the use of pomegranate for killing tape-worm is not new, the following items shows that it may be used in much larger quantity than usually.

Dr. Carl Bettelheim, of Vienna, narrates in the *Deutsches Archiv* a heroic method and nearly sure cure in the short space of three-quarters of an hour or two hours. It is this:

He inserts a tube in the œsophagus to the stomach, and pours down from two hundred to four hundred grams of very concentrated decoction of pomegranate root, having previously had his patient fast for twenty-four hours. The worm is stupified and passed, head and all to a certainty; the patient has no sickness of the stomach, and no nauseous swallowing to do; and the drug is cheap.—*Atlanta Med. and Surg. Jour.*

---

**Salicylic Acid in Erysipelas.**

Dr. S. W. Fowler, of Delaware, O., reports a number of cases of idiopathic erysipelas which were treated by the method first suggested by Dr. P. J. Spencer, of Cleveland, namely, by the internal and external use of salicylic acid. Five grains of the acid were administered every two hours, and the inflamed surface was washed every half hour, by means of a soft sponge, with the following solution:

℞

| | | |
|---|---|---|
| Acidi salicylici, ʒ ss. | or | 2.00 |
| Glycerinæ, fl. ℥ i. | | 35.00 |
| Aqua, fl. ℥ v. | | 150.00 |

After applying the solution, the entire inflamed surface was to be covered with cloths dipped in mucilage of elm bark.

The first application caused a peculiar burning sensation, growing less at each subsequent application. Quinine being likewise administered in small doses, recovery took place in about three days.—*Ohio Medical Recorder.*

**Hydrate of Chloral per Rectum.**

Dr. Starcke, having suffered extremely from loss of rest, in consequence of gastric catarrh, resorted to the use of chloral hydrate by enema, using about fifteen grains (one gram) thoroughly dissolved and passed high up into the rectum by means of a tube, in order to avoid irritation of the anus. The remedy produced most happy effects, since the doctor not only secured sound sleep, but gained at once in flesh and strength. The remedy was continued for some time in the same doses, but there was no difficulty whatever in relinquishing its use, as is so customary in cases where morphia is resorted to.—*Chicago Med. Jour. and Exam.*

---

**Hydrophobia Cured by Oxygen.**

This case is reported by Drs. Schmidt and Zebeden from Russia. The first symptoms of rabies appeared seventeen days after the injury. The patient was made to inhale three cubic feet of oxygen, and two hours afterwards he was in a state of perfect calm. Two days afterwards the symptoms of rabies reappeared, and another inhalation of oxygen was administered with the same success. This time the inhalation was continueed for forty-five minutes. A slight dyspnœa, which persisted after the disappearance of the graver symptoms, was treated for three weeks by the monobromide of camphor.—*Lyon Medical.*

---

**Chloral as a Counter-Irritant.**

Among the many uses to which chloral has been put, we have not met before with the following from the *Bulletin Therapeutique*: "Made into a mass with gum tragacanth, spread on paper and applied to the skin, it will produce a blister without pain. Applied as a powder, on cotton, it causes a painful, burning sensation. By the former method, a portion is absorbed and the patient falls asleep. Its action is not so uniform as cantharides, but as a mild vesicant, or an agreeable revulsive, the author quoted would commend such 'chloral paper' to physicians, the more so as it will keep for months without losing its activity, if well prepared."

---

**Phosphate of Zinc.**

Gros, in *La France Medicale*, extols this article, and advises its use in nervous affections, and especially in hysteria; giving at the same time a long list of neuroses in which it has been successfully used by physicians in America and England. He says that, though hysteria is an infection strange in its termination, so many cures have been reported that we should prefer this remedy to others because of its promptness of action, its facility of administration, and its innocuousness. It is stated that, contrary to expectation, it is innocuous, because if a toxic dose is given, vomiting invariably occurs, which prevents the poisonous action of the drug. The best form for administration is the granule.—*Medical Brief.*

---

**Combined Effects of Opium and Belladonna.**

Dr. J. N. Miller reports in the *British Medical Journal*, a case to prove that belladonna or its alkaloid rather intensifies than antidotes the effect of morphia.

In this case it was found that four-fifths of a grain of morphia, combined with one-fortieth of a grain of atropia, was equal in the relief of pain to two grains and two-fifths of morphia, and that it produced sound sleep at once, while the morphia alone gave only dozing and easily-interrupted sleep for an hour or more.

At another time, two doses of four-fifths of a grain of morphia and one-thirtieth of a grain of atropia were given with an interval of four hours, and had the effect of rendering the patient so comatose that it was only with the greatest difficulty that she was aroused.

---

**The Eucalyptus in Malarial Disorders.**

Dr. H. B. Dow, of London, contributes the following experience with this remedy, to the London *Lancet*:

In the first case in which I tried it, it was suggested to me by my patient, he saying that he had taken quinine by the pound without result, and that the eucalyptus was the only remedy for him. He had many years since contracted malaria of the worst type in the Douro district, and had tried most remedies without avail. A very few doses of the tincture of eucalyptus globulous removed the symptoms.

In the second case, my patient had been many years abroad as a missionary, and suffered severely from intermittent fever contracted during his labors in tropical climates. He also found no relief from quinine, but was very speedily relived by the eucalyptus.

In the third case, my patient was a gentleman who had lived many years in India and China, and during his residence abroad had had seven attacks of ague. Recently, he experienced a return of his old symptoms, and took quinine as he had been accustomed to, to check the illness, However, on this occasion it failed to produce the usual effect, so I recommended him to try the eucalyptus. The effect was at once marked, and speedily all his intermittent symptoms left him.

The remedy is pleasant to take, and the dose I have prescribed is ten minims of the tincture.

---

**Potassium Chlorate in Diphtheria.**

Dr. A. Seeligmueller regards a saturated solution of potassium chlorate as a veritable specific in diphtheria. He directs a tablespoonful of the following, nearly saturated, solution to be taken every hour, or every two hours :

℞

Potassium chlorate, 10 gm.
Water, 200, gm.

In the early stages of the disease, it is to be administered every hour, afterwards every two, and finally every three hours. Children under three years are to receive half a tablespoonful at a time. It is to be given both day and night, without interruption. Care should be exercised lest the remedy produce stomachic disturbances, or retard the heart's action. As soon as any symptoms appear which point to either of these effects, it should at once be suspended.— *Journ. de Therap. and L'Union Pharm.*

---

**Oxalate of Cerium and Caffein as Preventives of the Nauseating Effects of Opium.**

"For a long time I have been accustomed to prescribe a strong decoction of coffee, without milk or sugar, in drachm doses, administered every 15 minutes, to relieve the nausea and headache following the employment of opium. Since the introduction of the effervescing cerate of caffein, I have found it a very agreeable and effcient substitute for the less palatable beverage. These agents are, however, only applicable after the occurrence of the stomachic disturbances. The chirate of caffein, though less efficient than the oxalate of cerium, may be combined with opium. It is inadmissible in those cases where opium produces wakefulness instead of drowsiness. I have sometimes fancied that it lessened the sophorific without affecting the analgetic properties of opium. It may be that these applications of the drug are quite common, but if they have previously been published it has escaped my observation."—Dr. I. C. Busey, in *American Practitioner.*

**Morphia for Dysentery.**

Dr. Washington, of Augusta (Ga.,) in a desperate case of dysentery, the patient, in his agony of pain, being covered with cold, clammy sweat, the lips blue and cold, gave, by puncture, about the third of a grain of morphia, hoping for nothing more than to deaden the sensibility. In a few moments, to the surprise of the doctor, the sick man was perfectly quiet, and the vomiting and purging almost entirely relieved. Another puncture completed the work, and in less than forty hours he was convalescent. He afterwards treated satisfactorily a number of other cases by the same method, and finally himself. He was suffering severe vomiting and tenesmus, and found it necessary to be almost constantly on the stool. Opium, he tried, but could not retain it. Although so weak that he could not sit up, he then prepared his syringe, and gave himself a good puncture. "Lo ! in a few minutes he felt entire relief."

---

**Oxide of Zinc in Diarrhœa.**

Dr. Jacquier has followed, in the service of Dr. Bonamy at Nantes, the good effects of the employment of oxide of zinc in diarrhœa. The formula which he has employed is the following: Oxide of zinc, fifty-four grains; bicarbonate of soda, seven and a half grains; in four packets, one to be taken every six hours. In all the cases which he observed, oxide of zinc produced rapid cure of diarrhœa. In fourteen cases observed by Puygautier, the cure was even more rapid, since in only one case were three doses of the medicine required. The results are considered to have been more satisfactory, inasmuch as in several cases the malady had endured from one to many months, and other methods of treatment had not produced any improvement. Thus, he concludes that, although by no means to be held as exclusive treatment, the employment of oxide of zinc deserves to be more generally known as useful in diarrhœa—*British Medical Journal.*

---

**Warts for Skin Grafting.**

Dr. Ch. A. Seale, in the *Medical Record*, points out a new use for the unsightly cutaneous excrescence of warts. He says they can be employed with better results than small pieces of normal skin, in skin grafting, in consequence of being easily separated uninjured, into numerous cylindrical rods of great vascularity, and containing a large proportion of hypertrophied epithelium, which, when planted in healthy granulating tissue, readily adapt themselves to the new soil, receiving direct nourishment, and quickly growing as starting points for a new and smooth epithelial covering.

In one case, in which there had been complete destruction of all the skin on the dorsum of the foot, involving to a great extent the deep cellular tissue, and where for several weeks no healing advanced, grafts of freshly removed warts from the patient's hand immediately started little islands of new tissue, which rapidly increased until they coalesced and met the margins of the border skin, thereby completely covering the foot by a firm, protecting integument.

---

**Urethral Fever.**

Harrison, of Liverpool, refers to the fact that not unfrequently within a few hours after the passage of bougie, the patient experiences a sense of chilliness ; this rigor is usually followed by febrile excitement, which quickly subsides, without causing the patient much inconvenience or distress ; instances, however, occasionally occur when similar symptoms are speedily followed by death. These rarely occur when anæsthetics have been used, thus suggesting that the consciousness of pain may be an important factor in their production. A practical deduction to be drawn from this is : That too large a bougie should not be forced into the bladder. Where he has reason to fear a rigor will supervene after the introduction of an instrument, he invaribly prescribes a two minim dose of Flemming's tincture of aconite, to be given immediately after the operation. This he has found to be almost unfailingly effective. In the occurrence of a rigor he generally uses quinine in 5 to 10 grain doses, combining it with aconite, when there are early indications of febrile excitement.—*London Lancet.*

---

**Apomorphia for Clearing the Œsophagus.**

Dr. T. Verger was called to see a little girl who had just attempted to swallow a prunestone, which had lodged in the œsophagus. He made the child drink a little water, thinking that if this were swallowed some infusion of ipecac could be got into the stomach, with a view to causing the rejection of the foreign body by the efforts of vomiting. In vain! Not a drop could be got past the obstruction; all was rejected. At this moment a happy thought struck the doctor and his colleagues who were in consultation. "Try apomorphia." With the aid of a hypodermic syringe .0024 milligr. ($\frac{1}{30}$ gr.) apomorphia was injected under the skin of the thigh. Two minutes later an energetic attack of vomiting came on, during the first paroxysm of which the prune-stone was rejected. Some curious after-effects of the drug were observed. The child was seized with an irresistible desire to sleep, she could no longer remain standing, and the muscular sense was abolished. This condition lasted half an hour, notwithstanding the child was driven two kilometers in a carriage, and it only ceased after she had drank a cup of strong coffee.—*Bull. Gen. de Therap.*

---

**The Use of Iodine in Malarial Fevers.**

Since the publication of references to this subject (see *New Rem.*, pp. 234 and 298, 1878), a number of writers have contributed their experiences to various medical journals. J. H. Hervey, of Indianapolis, in the Cincinnati *Lancet and Clinic*, claims to have been using the remedy for the third of a century. He has never considered it a substitute for quinia in arresting the paroxysms of malarial fever, but finds it to be very serviceable in removing the various sequelæ and in preventing relapses. His custom is the use of the following formula:

℞

Tinctura iodinii,
Tinctura, ferri chloridi,
Tinct. sanguinariæ, āā equal parts.

13 to 15 drops to be taken after each meal for one to four weeks. This is used after the recurrence of the paroxysms has been arrested with quinia, and the latter is also continued in one-grain doses, before meals, for eight to ten days.

E. D. Laughlin, of Orleans (*Cin. Lan. and Clinic*), refers to the *Amer. Jour. of Med. Sci.*, Jan., 1875, in which is quoted an article by Dr. Munro, in which he claims to have tried iodine as an antiperiodic in 1869, in accordance with a suggestion of Willebrand.

Dr. Laughlin has, since 1875, used iodine in chronic ague with happy results, and also with good effect in typho-malarial fevers combined with other remedies. He prefers Lugol's solution to the tincture, giving ten to twelve drops thrice daily in ague, and five to eight drops every six hours in fever without regard to pyrexia. Its influence in reducing enlargement of the spleen is very decided.

In a subsequent number of the same journal,

J. N. Schell, M.D., of Frankton, Ind., says, that since the appearance of Dr. Fordyce Grinnell's article (see *New Rem.*, p. 298), he has used iodine in forty cases of quotidian and tertian types, and has had as good results with quinia. Some cases of quotidian ague were arrested at once, while two cases were not at all affected; he used the following formula:

℞

Tinct. iodinii, gtt. lx.
Potass. iodidi, gr. x.
Syr. simplicis, ℥ i.

A teaspoonful taken half an hour after a meal.

J. B. Day, M.D., of Evansville, Ind., writes to the *Lancet and Clinic* that during a scarcity of quinia, twenty or thirty years ago, he commenced the use of iodine as a substitute in cases of chronic intermittent fever, and has continued since to do so. He first employed it to reduce enlarged spleen, and then as an adjuvant in the treatment of chronic intermittents. "Scarcely any case, even under poor hygienic surroundings, will withstand a very persistent course for several weeks, daily, of iodine of iron." He never gives iodine in the acute form of the disease.

---

**The Form and Contagiousness of Yellow Fever.**

Mr. Robert Lawson, Inspector General of Hospitals, after a detailed consideration of the relation of this disease to other fevers, and especially of a number of cases which occurred on board H. M. S. Bristol, sums up his conclusions as follows:

1. Yellow fever is not a disease always presenting the continued form, but is met with frequently as a remittent, and even as an open intermittent.

2. The sporadic cases presenting yellowness of surface and black vomit are also found to have the train of urinary symptoms characterizing yellow fever, and are consequently identical with those met with during an epidemic.

3. In very many instances where persons in the vicinity of yellow fever cases are attacked with the disease, the facts do not admit of the exclusion of local causes, and such instances, therefore, cannot enable us to decide whether these causes or personal contagion have originated the disease; but from time to time other instances occur in which the exclusion of local causes can be assured, and in these, however extensive the exposure of susceptible individuals to the emanations from the sick may have been, the uniform result is that no communication of the disease has taken place.—*Lancet.*

---

**Harmlessness of Urea in the Blood.**

The London *Medical Record* mentions experiments by MM. Feltz and Ritter, to show that pure urea never brought on convulsive symptoms. Urea injected into the blood was eliminated very rapidly by the urine, and when it existed in considerable quantities in the organism it did not, as generally supposed, undergo a rapid transformation into carbonate of ammonia. Dogs into which urea was injected, after the renal vessels were tied, to prevent the rapid elimination of the poison, showed no more marked convulsive symptoms than others in which the same ligature was made without the injection. The convulsive symptoms observed with urea were produced by an impure substance containing ammoniacal salts. The authors summed up the following conclusions: 1. Pure urea, whether natural or artificial, injected into the venous system in large quantities, never brings on convulsive symptoms; it is rapidly eliminated by the secretions. 2. There are no ferments in the normal blood which convert the urea into ammoniacal salts. The rapidity of elimination cannot be regarded as the cause of this non-conversion, for by the suppression of the renal secretion the elimination of the urea may be retarded without accelerating the supervention of the eclampsia. The urea which in large doses brings on convulsions is always impure urea which contains ammoniacal salts, which are easily shown to be present by Nessler's reagent.—*Medical and Surgical Reporter.*

---

**Singular Plan of Counter-Irritation for Stings.**

The Southern darkey, to cure a toothache, will put a piece of red pepper in his eye. We had always taken literally his explanation, that he "soon done got enuff to do with dat eye, dat dar warn't no time to think of de tooth." But according to Dr. Lucas, of the Bombay army, the proceeding is rational, after all. This writer describes a similar treatment for scorpion stings. He says: "The sting is at first like a sharp prick from the point of a needle or a finely-pointed nail, and in a very few seconds it assumes a very agonizing form, as if innumerable pins and needles were thrust into the part. It then shoots up the limbs, along the course of the main nerve trunk, and is afterward of a darting and most excruciating nature, reaching its climax in from three to five minutes. When a pinch of powdered alum is put into the eye (the eye of the affected side being preferable), the pain of the reflected irritation ceases almost instantly as the conjunctival mucous membrane begins to smart; the local pain, perhaps less severe than before, gradually subsides after some hours, and its disappearance is, I am inclined to think, expedited by firm and steady pressure. In regard to the *modus operandi* of the alum cure, I will refrain from saying anything beyond that it probably acts by distracting pain and irritation elsewhere."—*Philadelphia Med. and Surg. Reporter*.

---

**Chromic Acid Test for Sugar in Urine.**

What is known as *Horsley's* test, consists of equal parts in solution of chromate of potash and liquor potassæ. If a freely alkaline solution of chromate of potash be mixed with urine supposed to contain sugar, and boiled, the sugar will assume a deep sap green color, arising from the decomposition of the chromic acid, the oxide of chromium being held in solution by the potash. The sensitiveness of this test is so great, that five or six drops only of saccharine urine, diffused through water, is sufficient to show the effect, which is far more striking than Trommer's or Moore's copper tests. If the quantity of sugar is very small, a piece of white paper placed at the back of the test tube will make the color more evident. With cane sugar no such effect is produced, only grape sugar undergoes the change of color.

Another test, known as *Luton's*, is extremely efficient, is most readily employed, easily prepared, and is unalterable. Its action requires no preliminary preparation of the urine, and it has succeeded often when other tests have failed. The presence of uric acid, urea, or albumen has no influence on the result. To a cold saturated solution of bichromate of potash, add sulphuric acid in excess, so that free sulphuric acid may be present after the liberation of all the chromic acid. The solution is, therefore, composed of water, chromic acid, bisulphate of potash, and an excess of sulphuric acid, and has a beautiful red color. Add the test liquid to the diabetic urine, until you get a strong tint of red. Apply the spirit lamp, when, a brisk effervescence ensuing, the color changes from red to emerald green. The chromic acid yields oxygen to the sugar, and carbonic acid, water, and sesquioxide of chrome result, which last, uniting with the acid, forms a persulphate of the sesquioxide of chrome.

---

**Ergotine in Acute Ophthalmia.**

Dr. Planat, of Nice, has found ergotine act with efficacy and promptitude in proportion as oculo-palpebral phlegmasiæ are simply inflammatory. In blepharo-conjunctivitis the improvement is first observed in the conjunctiva; and in keratitis, although still very active, it is a degree less so than in the more superficial affections. It is also of great service in iritis, rapidly subduing the acute manifestations and preventing their extension to the external membranes of the eye. When these last are the seat of a chronic fluxion dependent on a scrofulous or dartrous diathesis, ergotine, without influencing the constitutional affection, acts none the less efficiently on the inflammatory element—a fact of importance, as by generally preserving the eye from plastic deposits, corneal ulcers, and consecutive staphylomas, it allows of the treatment for the diathesis being more promptly put into force. The formula which Dr. Planat recommends is from one to one and a half gramme of ergotine in twenty of glycerine or rose water, of which from eight to ten drops are to be inserted in the eye every two hours. Where there is violent inflammation of the eyelids or distention of the conjunctiva, a rag wetted in this mixture should be left on the parts for some hours. In general, two or three days suffice for the subdual of the most intense blepharo-conjunctivitis. Dr. Planat has employed the ergotine in this way, with invariable success, for several years past.—*Journal de Therap.*

---

**Oxalate of Cerium in Chronic Cough.**

A Medical writer in the *Medical Record* says: I have noticed in several of the current medical periodicals, testimonials as to the efficacy of oxalate of cerium in chronic coughs. I desire, through the medium of your valuable journal, briefly to add my own experience in its use.

My attention was first called to a drug, in the capacity already mentioned, something over a year ago, since which time I have used it in a goodly number of instances, until I have come to regard it as one of the principal remedies in the treatment of this distressing malady.

Coughs resulting from chronic bronchitis, phthisis, and chronic laryngitis, have promptly yielded to this remedy in my hands, after both the internal and external administration of other drugs had signally failed. In giving it to adults, in only one instance have I ever experienced any ill effects from its use. This was a case very similar to the one reported in the *Record* for December 28, 1878, by Dr. La Roe, of Greenpoint, L. I. On this occasion I used seven grains at first, which produced the narcotic effect he speaks of as following the taking of five grains. I then reduced the quantity to five grains, which quieted the cough without the deleterous effects first produced. When I first began using oxalate of cerium, I produced decided narcotic effects in two instances from the administration of five grains to children of from ten to thirteen years of age.

---

**Whooping Cough.**

Dr. M. T. Dellenbaugh, of Philadelphia, writes to the *Medical Times*, as follows:

A SPECIFIC FOR WHOOPING-COUGH.—*Dear Sir.*—I write you to say that I believe I have succeeded in using a specific for whooping-cough.

Believing that whooping-cough is the result of the location of a specific contagium on or in the mucous membrane of the respiratory apparatus, from my experience with the muriate and carbonate of ammonia I was of the opinion that the beneficial results obtained by their use in the treatment of bronchitis could be ascribed to a stimulent effect on the respiratory mucous membrane, as the ammonia is eliminated from the system through this membrane. In looking over the list of antizymotics, I rejected all the familiar preparations as most likely useless, or they would have been found useful long since. I concluded that to subserve the purpose indicated, I must have an antizymotic that will be eliminated by the respiratory mucous membrane, and the only thing likely to answer the purpose would be the picrate or carbazotate of *ammonia.*

I have treated six children suffering with whooping-cough (at the Howard Dispensary,) in accordance with the above related conclusions, and, I am very happy to state, with the most gratifying results. The mothers assured me that after their children would take two or three doses of the medicine the paroxysm would relax in severity, and in a couple of days would entirely subside as a paroxysmal cough with the well-known whoop, and a simple cough of ordinary laryngo-bronchitis remain. I gave the remedy in this way:

℞

Picrate of ammonia, gr. i.
Muriate of ammonia, gr. XXIV.
Powdered extract of liquorice, ℥i.
Water, fl. ℥iii.

M.—S. Teaspoonful every 3 hours to a child 6 months and under, doubling the quantity for a child of about one year or two years of age, and giving as much as ¼ gr. to a child 3 to five years of age.

It might be a coincidence that these six children (belonging to three different families), recovered under the use of the medicine, but I, of course, believe it to be solely the result of the picrate of ammonia. My opportunity for observing whooping-cough in private practice is very limited. Knowing that you are specially interested in therapeutics, I would like you to submit the treatment I have suggested to a test, provided it impresses you favorably.

I have requested several of my friends in the profession to give it a trial. So far as I know, the treatment of whooping-cough heretofore has been quite discouraging, especially to parents, and I am *very* sure there would be no harm done or time lost by the treatment with picrate of ammonia.
*Medical Times.*

---

**Ointment of Nitrate of Silver.**

It is said that vaseline forms a good base for making an ointment with nitrate of silver.—Whether such an ointment will find much application as a remedy may be doubted; but we should judge it to be a very good pomade for dying the hair and beard provided it keeps as well as has been alleged.

---

**Batiator Root, A Substitute for Ipecac.**

(Stanislas Martin.) The author gives a description of this root, which is derived from a plant growing in Senegal. Seeds of it have been planted in the Museum of Natural History at Paris, from which the plant will hereafter be determined. The root is identical in its effects with the ipecac, in the same doses.—*L'Union Pharmaceutique.*

